# Treatment outcome and factors affecting time to recovery in children with severe acute malnutrition treated at outpatient therapeutic care program

**DOI:** 10.3402/gha.v9.30704

**Published:** 2016-07-08

**Authors:** Melkamu Merid Mengesha, Negussie Deyessa, Balewgizie Sileshi Tegegne, Yadeta Dessie

**Affiliations:** 1 Department of Public Health, College of Health and Medical Sciences, Haramaya University, Harar, Ethiopia; 2 Department of Preventive Medicine, School of Public health, Addis Ababa University, Addis Ababa, Ethiopia

**Keywords:** time to recovery, severe acute malnutrition, outpatient therapeutic care, health post, Southern Ethiopia

## Abstract

**Background:**

The outpatient therapeutic care program (OTP) of children with severe acute malnutrition (SAM) has been decentralized to health post level in Ethiopia since 2008–2009. However, there is a lack of evidence regarding treatment outcomes and factors related to the duration of stay on treatment after its decentralization to health post level.

**Objective:**

This study was aimed to assess treatment outcome and factors affecting time to recovery in children with SAM treated at OTP.

**Design:**

Health facility–based retrospective cohort study was conducted using data from 348 patient cards. The outcome variable was time to recovery. Descriptive analysis was done using percentages for categorical data and mean/median for continuous variables. A robust method of analyzing time to event data, the Cox proportional-hazard regression, was used. All statistical tests in this study are declared significant at *p<*0.05.

**Result:**

89.1% of children with kwashiorkor and 69.4% of children with marasmus were recovered. Of the total children studied, 22% were readmitted cases. The median time of recovery was 35 days for children with kwashiorkor and 49 days for children with marasmus. Children older than 3 years were 33% less likely to achieve nutritional recovery [adjusted hazard ratio, AHR=0.67, 95% confidence interval, CI (0.46, 0.97)]. Similarly, marasmic children stayed longer on treatment [AHR=0.42, 95% CI (0.32, 0.56)]. However, children who gained Mid-Upper Arm Circumference (MUAC) ≥ 0.24 mm/day were 59% more likely to recover faster [AHR=1.59, 95% CI (1.23, 2.06)].

**Conclusions:**

Close monitoring of weight and MUAC gain to assess nutritional improvement with due emphasis given to children with lower admission weight, children of age 3 years and above and marasmic children will have a positive effect on treatment duration and outcome.

## Introduction

Globally, it is estimated that there are nearly 20 million children who are severely acutely malnourished. Most of them live in South Asia and Sub-Saharan Africa (1). Severe acute malnutrition (SAM) is defined by weight for height <−3 standard deviation or by Mid-Upper Arm Circumference (MUAC) value of less than 110 mm in children aged 6–59 months (1, 2). However, instead of using MUAC<110 mm, the World Health Organization (WHO) guideline updates on the management of SAM strongly recommended the use of MUAC<115 mm to identify children with SAM (3). Evidences indicate that the risk of mortality in acute malnutrition is directly related to severity (4, 5). Worldwide, there are about 1.5 million child deaths associated with severe wasting and 3.5 million deaths associated with moderate wasting every year (4, 5). In Ethiopia, the 2011 Ethiopian demographic and health survey (EDHS) reported a remarkable decline in under-five mortality, from 166 per 1,000 in the year 2000 to 88 per 1,000 in 2011 (6). However, the prevalence of wasting in Ethiopia has remained constant over the last 11 years (6).

The treatment of children with SAM was previously limited to inpatient therapeutic feeding centers (7). However, the innovation of ready-to-use therapeutic food (RUTF), use of MUAC both for screening and admission, and the new classification scheme of SAM made treatment possible at the community level (7, 8). In the outpatient therapeutic care programs (OTPs) of different resource-limited settings, the identification and treatment of children with SAM was effectively done by community health workers (1, 9, 10). In this system of care, children with SAM attend an OTP site weekly to receive RUTF to eat at home and a course of routine medications including amoxicillin, vitamin-A, measles, and deworming (2, 11). RUTF is a lipid peanut paste that resists bacterial contamination, contains very little water, does not require cooking, is energy dense (23 kJ/g), and meets the compositional requirement specified by the WHO for therapeutic food (12).

Studies conducted in Ethiopia and other settings have documented the effectiveness of community-based management of SAM regarding treatment outcomes and coverage even though most of them were conducted during the emergency setting (7, 13–15). In the developmental setting, the community-based management of SAM has been decentralized to health post level in Ethiopia since 2008–2009 (16). As there are no studies in the post-emergency setting, there is a lack of evidence concerning the magnitude of treatment outcome, duration of stay on treatment, and related factors at health post level in the country. Therefore, this study was conducted to fill in the gap with the specific objectives of assessing the magnitude of treatment outcome, comparing the median times of recovery among children with kwashiorkor and marasmus, and identifying predictors of time to recovery.

## Materials and methods

### Setting and study design

The study was conducted in Shebedido woreda (district), Southern Ethiopia. Shebedino is one of the 21 woredas found in Sidama zone. It is located 27 km away from the capital city of the southern nations, nationalities and peoples (SNNP) region, Hawassa. The woreda has a total of seven health centers and one district hospital. Under each health center, there were five health posts (the lowest primary health care unit) staffed by two or three female health extension workers (HEW). When this study was conducted, there were a total of 32 health posts delivering OTP service in the rural kebeles (villages) of the woreda. Staffs of the health posts were grade 10 complete and received 1-year training on the health extension program module (17). They were also trained to manage children with SAM without medical complications (18). Based on the protocol for the management of children with SAM, HEWs screen children with SAM for admission in the program, provide routine medications, monitor the progress of the children, and refer children with complications or those who fail appetite tests to inpatient care (19).

We conducted a retrospective cohort study using the data of severe acutely malnourished children admitted to 12 of the available health posts in Shebedino district between January 1, 2011, and January 1, 2013.

### Participants

The study was conducted through record review of eligible patient cards of children who were treated for SAM in the selected health posts. The patient cards of children that fulfilled the following criteria were reviewed: children of age between 6 and 59 months, children who received treatment between January 1, 2011, and January 1, 2013, children with admission MUAC value <110 mm or bilateral pitting nutritional edema, children who passed the appetite test, and those children with no medical complications. However, the study excluded children whose patient cards indicated that they were referred to OTP after being refused inpatient admission and who were stabilized in and referred from a therapeutic feeding center. Study participants were dichotomized into two cohorts based on the presence of nutritional edema (marasmic cohort: children who had no edema; and kwashiorkor cohort: children who had edema).

### Sample size

We used Open Epi version 2.3 (20) to calculate the sample size with the following assumptions: The proportion recovered in the exposed (children with marasmus) group (81%), the proportion recovered in the non-exposed (children with kwashiorkor) group (96%) (21), 95% CI (confidence interval), 5% marginal error (d), and power of 80%. Accordingly, the minimum sample size calculated for each group was 85. We used a design effect of two to compensate for potential losses during multi-stage sampling and added 10% of the sample for missing and incomplete data. The final sample size obtained was 374 with a sample size of 187 for each of the cohorts in a one-to-one ratio.

### Sampling technique

**Fig. 1:**
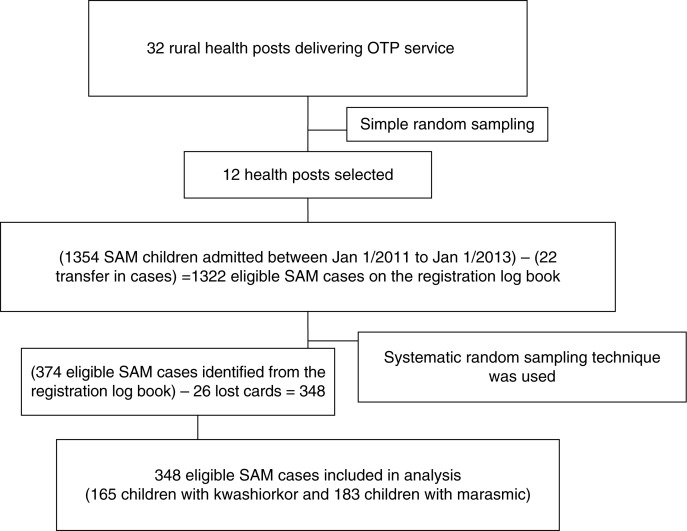
Sampling scheme of severe acutely malnourished children included in the study. Simple random sampling was used to select health posts. In the selected health posts, systematic random sampling was used to select eligible SAM children. SAM refers to Severe Acute Malnutrition and OTP refers to Outpatient Therapeutic Care Program.

Shebedino woreda has a total of 32 rural kebeles with the ratio of one health post per kebele. And each health post had a catchment population of 5,000. Therefore, we assumed homogeneity among the kebeles in terms of access to the health facility, population size, infrastructure availability, and agricultural production. With this assumption, we decided to take a random sample of 12 health posts. From the selected health posts, eligible children with SAM were identified from the registration log book and sampling frame was developed per OTP site. The total sample size was then proportionally allocated to the selected health posts. Finally, from each health post, eligible study subjects were selected using systematic random sampling (Fig. 1).

### Data collection and quality

The HEWs collected the data between January 31 and February 28, 2013, using structured questionnaire to extract information from patient card. Both the data collectors and the supervisor were given 2 days of training. As part of the training, the data collection tool was pretested before the actual data collection to maintain data quality. The information collected included the socio-demographic characteristics of each child including age, sex, OTP site; patient baseline information including date of admission, type of admission, admission anthropometry, and routine admission medication; follow up information on weight, MUAC, edema, Plumpy-nut provided per week, appetite test, treatment outcome, follow up date, and date outcome ascertained. Baseline admission characteristics in terms of medications received, season of admission, type of admission, and breast feeding are shown in Table 1. Body weight was measured using a digital weighting scale or by a 25-kg hanging spring scale graduated by 0.1 kg for children below the age of 3 years. Similarly, MUAC was measured on the left upper arm of a child with the arm hanging down the side of the body and relaxed. The MUAC value was recorded to the nearest value of 1 mm. Appetite test was conducted every week in a quite environment on each visit for child enrolled in the program. A child was said to pass the appetite test when she or he was able to consume the amount of RUTF recommended for her or his body weight shown in Table 2 (2, 19). Children who failed the appetite test in any visit are referred to inpatient care (2, 19). According to the national protocol, children receive different number of RUTF (500 kcal/sachet) sachets weekly to eat at home depending on their weight (2, 19). Admission medication included amoxicillin given on admission and for seven consecutive days, vitamin A given on the date of admission and on the fourth visit, measles vaccine on the fourth visit, and deworming on the second visit. Vitamin A was not given on admission for children with edema and for those who received vitamin A in the past 6 months.

**Table 1:** Baseline admission characteristics of SAM children admitted to the selected health posts between January 1, 2011, and January 1, 2013, in Shebedino woreda, Southern Ethiopia

Variables	Kwashiorkor (*n*=165)	Marasmus (*n*=183)	Total
Season of admission (*N*=348)			
Summer	57 (34.5)	71 (38.8)	128
Autumn	25 (15.2)	40 (21.9)	65
Winter	8 (4.8)	16 (8.7)	24
Spring	75 (45.5)	56 (30.6)	131
Admission (*N*=318)			
New	114 (78.6)	134 (77.5)	248
Readmission	31 (21.4)	39 (22.5)	70
Medication received (*N*=339)			
Amoxicillin only	25 (15.2)	37 (20.2)	62
Amoxicillin and deworming	33 (20.0)	21 (11.5)	54
Amoxicillin and vitamin A	11 (6.7)	12 (6.6)	23
Amoxicillin and measles	5 (3.0)	2 (1.1)	7
Amoxicillin, deworming, and vitamin A	15 (9.1)	16 (8.7)	31
Amoxicillin, deworming, and measles	36 (21.8)	na†	36
Amoxicillin, vitamin A, and measles	7 (4.2)	33 (18)	40
Amoxicillin, deworming, vitamin A, and measles	24 (14.5)	62 (33.9)	86
Breast feeding status on admission (*N*=339)			
Yes	22 (13.8)	72 (40.9)	94
No	138 (86.2)	107 (59.1)	245

† Not available. SAM=severe acute malnutrition.

**Table 2:** The amount of Plumpy nut a child is expected to consume during the appetite test

Body weight in kilogram	Plumpy nut sachet the child expected to consume
<4	1/8–1/4
4–10	1/4–1/2
11–15	1/2–3/4
>15	3/4–1

Completed questionnaires were collected on daily basis and checked for completeness and consistency. Cleaning was done on daily basis and timely feedback was communicated to the data collectors.

### Measurement of variables

**Fig. 2:**
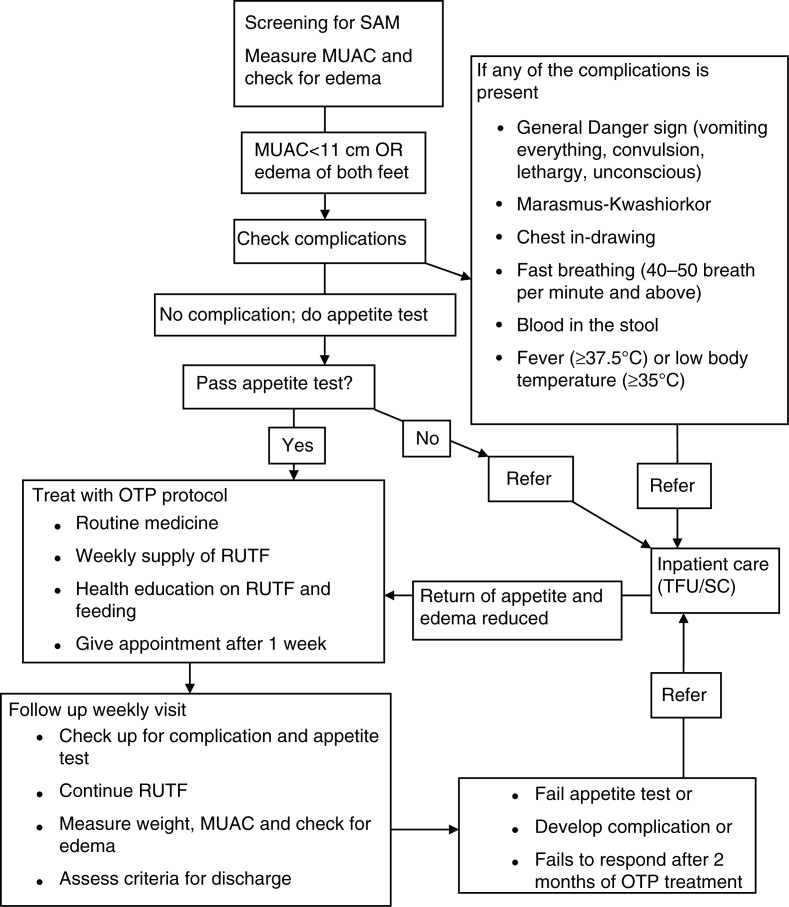
OTP treatment protocol used in the study setting. Children who had MUAC value <11 cm or had edema and had no medical complications and passed appetite test were admitted to OTP. Children in the OTP had weekly follow up for medical checkup, RUTF supply, and anthropometric measurements. Those who developed complications or failed to respond to treatment were referred to inpatient care.

The rate of MUAC gain in mm/day and weight gain in g/kg/day was calculated for both marasmic children and children with kwashiorkor. For children with kwashiorkor, the rate of MUAC gain and weight gain was calculated after the edema has resolved. Admission type to OTP refers either to new admission (admitted for the first time or after 2 months of recovery) or readmission (admitted within 2 months of recovery). A child may be discharged from OTP by recovery, transfer out, default, death, or non-response. Recovery was defined when a child attained 15% weight gain (target weight) and had no edema for two consecutive weeks or visits. A child was said to be non-responsive to treatment if she or he failed to recover at the end of the eighth week of treatment in the program. Children who failed to respond after 2 months of treatment in the OTP are referred to inpatient care (19). Fast recovery refers to the nutritional recovery of a child before the eighth week of treatment in the OTP. When a child was absent for three consecutive follow-up visits from the OTP and confirmed by home visit, she or he was classified as a defaulter. Complications refer to failed appetite test, lower respiratory infection, severe generalized edema, marasmic kwashiorkor, severe dehydration, and high fever (Fig. 2) (2, 19).

### Analysis

Data were entered into Epi-Data for windows version 3.1 (version 3.1, EpiData Association, Odense, Denmark) and analyzed using statistical package for social sciences (SPSS) for windows version 16.0 [Release 16.0.0, September 13, 2007, Copyright (c) SPSS, Inc., 1989–2007]. Descriptive analysis was done using percentages for categorical data and mean/median for continuous variables. Pearson chi-square test was used to see associations between categorical variables. The Mann–Whitney U-test for the independent two-sample test was used to compare the medians. The Kolmogrov–Smirnov test of normality was used to check the normality of distributions for continuous variables. The dependent outcome variable was time to recovery (i.e. the time it takes for a malnourished child to attain nutritional recovery). Individuals were excluded if they transferred out to inpatient care due to complications and classified as non-responsive after 2 months of treatment at OTP. Treatment outcome was dichotomized into excluded and recovered. The Kaplan–Meier product limit, life-table analysis, and log-rank tests were used to estimate the time to recovery and the cumulative proportion surviving in a given interval, and compare the survival curves, respectively. The Cox proportional-hazard regression was used to identify predictors of time to recovery. Co-linearity was checked for the covariates in the final model and the proportional hazards assumption was checked using STATA for windows version 11 (Version 11.0, copyright 1984–2009 StataCorp, TX, Serial number: 40110523523). All statistical tests in this study were declared significant at *p*<0.05.

### Ethical issues

Ethical approval was obtained from the Institutional Review Board (IRB) of Addis Ababa University, School of Public Health, with reference number SPH/266/05. As the study was conducted through a review of records, no consent was obtained from the mothers or caregivers of the study subjects. No personal identifiers were used to collect the data to maintain confidentiality.

## Results

A total of 348 patient cards were included in the review and there were 26 lost patient cards (22 from the kwashiorkor group and 4 from the marasmic group). Therefore, the analysis was based on data obtained from 88.2% (165/187) of patient cards from the kwashiorkor group to 97.9% (183/187) from the marasmic group. Females constituted 55.4% (185/334) of the children studied. The overall median (interquartile range, IQR) age was 36 (24, 48) months with 48 months (36, 48) for children with kwashiorkor and 36 months (12, 48) for children with marasmus.

The overall proportion of recovery was 78.7% with 89.1% for children with kwashiorkor and 69.4% for children with marasmus. Among children with marasmus, 28.4% (52/183) did not respond to treatment. A total of 4.9% (17/348) cases were referred to inpatient due to complications during the follow-up visits. Referred cases among children with kwashiorkor and children with marasmus were 7.9% (13/165) and 2.2% (4/183), respectively. Of the total admissions to the program during the study period, 22% (70/318) were readmitted cases. The median (IQR) rate of weight gain among recovered children was 3.85 g/kg/day (3.29, 4.66) in children with marasmus and 4.45 g/kg/day (2.75, 6.21) in children with kwashiorkor. Similarly, the overall median (IQR) rate of MUAC gain was 0.25 mm/day (0.16, 0.41) with 0.24 mm/day (0.14, 0.43) for children with kwashiorkor and 0.26 mm/day (0.18, 36) for children with marasmus (Table 3). The discharge MUAC value for recovered children was between 11 and 11.5 cm, 11.6 and 12.4 cm, and 12.5 cm or more for 20.6% (56/272), 42.6% (116/272), and 36.8% (100/272), respectively.

**Table 3:** The distribution of patient follow-up characteristics among children with SAM admitted to OTP in Shebedino woreda, Southern Ethiopia, from January 1, 2011, to January 1, 2013

Variables	Kwashiorkor (*n*=165)	Marasmus (*n*=183)	χ^2^ (df)	*p*
Outcome (*N*=348)				
Recovered	147 (89.1)	127 (69.4)	44.17 (2)	<0.001*
Non-response	5 (3)	52 (28.4)		
Transfer out	13 (7.9)	4 (2.2)		
Weight gain (g/kg/day) (*N*=335)				
Median (IQR)	4.45 (2.68, 6.22)	3.46 (2.83, 4.35)	10998.5†	0.001*
Kolmogrov–Smirnov *Z*=2.66				<0.001‡
Median (IQR) among recovered	4.45 (2.75, 6.21)	3.85 (3.29, 4.66)		
Overall median (IQR)	3.72 (2.75, 5.41)			
Discharge MUAC (cm), (*N*=337)				
Median (IQR)	12.5 (11.5, 13)	11.7 (11.5, 12.0)	6,755†	<0.001*
Kolmogrov–Smirnov *Z*=4.13				<0.001‡
Overall median (IQR)	12 (11.9, 13)			
Discharge weight (*N*=343)				
Median (IQR)	11 (9.7, 12.7)	8.5 (6.7, 11.5)	7,915†	<0.001*
Kolmogrov–Smirnov *Z*=3.74				<0.001‡
Overall mean (SD)	10.08 (±2.79)			
MUAC gain (mm/day), (*N*=328)				
Median (IQR)	0.24 (0.14, 0.43)	0.26 (0.18, 0.38)	12,649†	0.432
Kolmogrov–Smirnov *Z*=1.70				0.01‡
Overall median (IQR)	0.25 (0.16, 0.41)			

* Significant at *α*=0.05

† the Mann–Whitney U-test of two independent samples test was used

‡ *p*-value for K–S test. IQR=interquartile range; SAM=severe acute malnutrition; OTP=outpatient therapeutic care program.

**Fig. 3:**
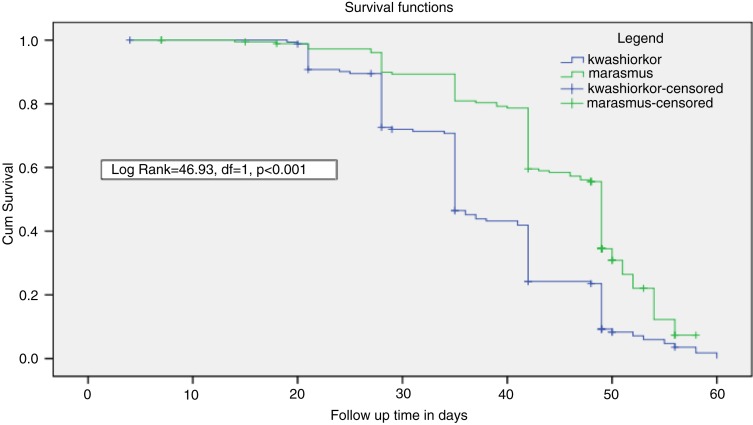
Comparison of survival curve for children with kwashiorkor and marasmus treated at OTP.

The overall median time of recovery was 42 days with 35 days for children with kwashiorkor and 49 days for children with marasmus. The survival curve for the two cohorts showed a statistically significant difference (log rank=46.93, df=1, *p<*0.001; Fig. 3). The cumulative proportions of children surviving recovery at the end of the seventh week of treatment in the kwashiorkor and marasmic cohorts were 23% (39) and 55% (100) cases, respectively (Table 4). The overall incidence rate of recovery with 95% CI was 14.92 (13.21, 16.8) children per 100 person weeks observed. The incidence rates of recovery were 18.54 (15.66–21.79) and 12.18 (10.15, 14.49) among the kwashiorkor and marasmic cohorts, respectively. There was no difference in the recovery times between the two cohorts for a weight gain rate of ≥3.21 g/kg/day (Log rank=2.84, df=1, *p=*0.092). In the bivariate Cox regression model admission weight, the rate of MUAC gain and the type of SAM were found to be significant factors affecting recovery time (Table 5). The covariates having *p*<0.1 in the bi-variate Cox regression model were entered in to the multivariate Cox Regression model. For clinical significance and control of confounding, age, sex, medication, and season of the year were included in the multivariable Cox regression. Controlling for the effects of admission weight, sex, medication, and season of the year; the age, type of SAM, and rate of MUAC gain (mm/day) were independently associated with the time to recovery. Children older than 3 years were 33% less likely to achieve nutritional recovery compared to the reference [adjusted hazard ratio, AHR=0.67, 95% CI (0.46, 0.97)]. Similarly, children with marasmus stayed longer on treatment compared to children with kwashiokor [AHR=0.42, 95% CI (0.32, 0.56)]. However, children who gained MUAC ≥0.24 mm/day were 59% more likely to recover faster [AHR=1.59, 95% CI (1.23, 2.06)] (Table 6).

**Table 4:** The life table analysis of severely acutely malnourished children treated at outpatient therapeutic care program in Southern Ethiopia from January 2011 to January 2013

Nutritional status	Time interval in days	Number of entering intervals	Number of withdrawals during intervals	Number of subjects exposed to risk	Number of subjects recovered	Proportion not recovering	Cumulative proportion not recovering
Kwashiorkor	0–7	165	1	164.5	0	1.00	1.00
	7–14	164	1	163.5	0	1.00	1.00
	14–21	163	1	162.5	2	0.99	0.99
	21–28	160	2	159	15	0.91	0.89
	28–35	143	2	142	30	0.79	0.71
	35–42	111	2	110	45	0.59	0.42
	42–49	64	3	62.5	28	0.55	0.23
	49–56	33	5	30.5	24	0.21	0.05
	56–63	4	1	3.5	3	0.14	0.01
Marasmus	0–7	183	0	183	0	1.00	1.00
	7–14	183	3	181.5	0	1.00	1.00
	14–21	180	2	179	2	0.99	0.99
	21–28	176	0	176	5	0.97	0.96
	28–35	171	0	171	12	0.93	0.89
	35–42	159	0	159	19	0.88	0.79
	42–49	140	4	138	41	0.70	0.55
	49–56	95	44	73	46	0.37	0.20
	56–63	5	3	3.5	2	0.43	0.09

**Table 5:** Factors affecting time to recovery in the bivariate Cox regression in children with SAM treated at OTP in Shebedino woreda, Southern Ethiopia, from January 1, 2011, to January 1, 2013

Variables	Frequency	Recovered, *n* (%)	CHR (95% CI)
Age (in months)			
<36	113	84 (74.3)	1
≥36	235	190 (80.9)	1.12 (0.84, 1.44)
Sex			
Male	149	116 (77.9)	1
Female	185	145 (78.4)	1.08 (0.84, 1.38)
Admission type			
New	248	190 (76.6)	0.88 (0.66, 1.18)
Readmission	70	60 (85.7)	1
Type of SAM			
Kwashiorkor	165	147 (89.1)	1
Marasmus	183	127 (69.4)	0.48 (0.38, 0.61)*
MUAC gain (mm/day)			
<0.24	154	114 (74.0)	1
≥0.24	174	154 (88.5)	1.48 (1.16, 1.89)*
Medication			
Amoxicillin plus‡	277	219 (79.1)	0.92 (0.67, 1.26)
Amoxicillin	62	47 (75.8)	1
Season			
Summer and autumn	193	152 (78.8)	1
Winter and spring	155	122 (78.7)	1.12 (0.88, 1.43)
Admission weight			
<6.5	76	49 (64.5)	1
≥6.5	272	225 (82.7)	1.53 (1.12, 2.08)†

* *p*<0.001

† *p*<0.01

‡ amoxicillin plus included amoxicillin, vitamin A, measles vaccine and deworming. CHR=crude hazard ratio; CI=confidence interval; SAM=severe acute malnutrition; OTP=outpatient therapeutic care program.

**Table 6:** Predictors of time to recovery in the multivariate Cox Regression in children with SAM treated at OTP in Shebedino woreda, Southern Ethiopia, from January 1, 2011, to January 1, 2013

Variables	CHR (95% CI)	AHR (95% CI)
Type of SAM		
Kwashiorkor	1	1
Marasmus	0.48 (0.38, 0.61)	0.42 (0.32, 0.56)*
MUAC gain (mm/day)		
<0.24	1	1
≥0.24	1.48 (1.16, 1.89)	1.59 (1.23, 2.06)*
Age in months		
<36	1	**1**
≥36	1.12 (0.86, 1.44)	0.67 (0.46, 0.97)‡
Sex		
Male	1	1
Female	1.08 (0.84, 1.38)	1.19 (0.93, 1.53)
Season of the year		
Summer and autumn	1	1
Winter and spring	1.12 (0.88, 1.43)	1.14 (0.89, 1.47)
Admission weight		
≤6.5	1	1
>6.5	1.53 (1.12, 2.08)	1.31 (0.84, 2.05)

* *p*<0.001

‡ *p*<0.05. CHR=crude hazard ratio; CI=confidence interval; AHR=adjusted hazard ratio; SAM=severe acute malnutrition; OTP=outpatient therapeutic care program.

## Discussion

Children who achieved nutritional recovery were 78.7% with a median recovery time of 42 days. Marasmic children stayed longer on treatment compared to children with kwashiorkor. The factors identified to affect time to recovery were type of SAM, age, admission weight, and the rate of MUAC gain. The proportion of recovery noted in this study has met the minimum SPHERE standard which recommends >75% of recovery from therapeutic care (22). The reported proportion of recovery in this study was consistent with findings from different settings (10, 12, 23, 24). However, a higher proportion of recovery was also reported from a study done in Bedawacho, Southern Ethiopia, and Burkina Faso (21, 25). The proportion of non-response to treatment was higher in this study compared to 8.91% in Tigray and 8% in Wollo (12, 24). The difference against the Wollo setting may be explained by the use of a longer time period to define non-response to treatment (12). In the Tigray study, however, children who received treatment at health center and health post were included in the study (24). Consequently, because of referrals from health posts, children who receive treatment at health center were likely to have medical complications and may get more attention which may have contributed to better outcome. Furthermore, according to a study in Burkina Faso, the discharge criteria of 15% weight gain may have also contributed to the higher non-response noted in this study by giving the studied children shorter period to attain nutritional recovery (25).

Of the total admissions to the OTP, 22% were readmitted cases. First, the observed high proportion of readmission may be due to lack of a supplementary feeding program during the time for which data was obtained. Second, it may be because of an early discharge associated with the use of 15% weight gain criteria (25). For example, in this study, 20.6% of children recovered had MUAC value between 11 and 11.5 cm up on discharge. If the newly WHO recommended MUAC cutoff value (3) for admission, <11.5 cm, was implemented in the study setting to define SAM, 20.6% of recovered children had still been classified as SAM. Therefore, in the study setting, more children were still being declared cured without having attained an adequate nutritional recovery, which may in turn lead to an increase in readmitted cases.

The median time of recovery reported in this study was consistent with findings from the Southern and Northern Ethiopia (21, 24). It was also beyond the minimum SPHERE standard for treatment duration of children with SAM at OTP (22). The acceptable length of stay for outpatient management of SAM according to the minimum SPHERE standard was <4 weeks for a weight gain rate of ≥8 g/kg/day (22). Regarding the factors associated with time to recovery, children with marasmus were found to stay longer on treatment and this was consistent with a study in Malawi, in which SAM children without edema were less likely to recover [hazard ratio, HR=0.80, 95% CI (0.69–0.94)] (26). This same study has also reported that lower enrollment value for weight was related to treatment failure (26), which supported our finding that, for all children, an admission weight of greater than 6.5 kg was associated with a higher likelihood of recovery compared to weights below 6.5 kg. However, this association in the bivariate was lost in the multivariable Cox regression.

The strength of this study was that we used time to event data and a robust method of analysis to identify important factors affecting the time to recovery of SAM children treated at health post-level operating in rural kebeles. However, the limitations of the retrospective data, including the incompleteness or loss of data, were evident in this study. Additionally, this study did not assure that RUTF was not shared by other siblings in the household or that the sick child was fed appropriately, and it also did not assess the household and environmental factors, and the socio-economic and demographic characteristics of the care taker.

## Conclusions

Proportion of recovery has met the minimum SPHERE international standard for the outpatient treatment of SAM. However, the treatment duration was longer than the SPHERE recommendation. Factors identified as positively affecting the time to recovery were admission weight and the rate MUAC gain. Alternatively, the age and type of SAM affected time to recovery negatively. Therefore, the close monitoring of weight and MUAC gain, to assess nutritional improvement – making note of the distinctions among children who have lower admission weights, are older, and suffer from different kinds of SAM – will have a positive effect on treatment duration and outcome. Finally, for further investigation, we suggest a prospective cohort study on problems related to non-response to treatment and readmission.

## Paper context

OTP was reported to be effective in minimizing the rate of defaulter and death cases among children with SAM in studies conducted during the emergency setting. However, in the developmental setting, the rate of nutritional recovery of SAM children and factors affecting it are not well assessed in Ethiopia. Therefore, to fill in this gap, this study was conducted with the aim to assess treatment outcome and factors affecting time to recovery in children with SAM treated at OTP.
